# In Vivo Imaging Biomarkers in Mouse Models of Alzheimer's Disease: Are We Lost in Translation or Breaking Through?

**DOI:** 10.4061/2010/604853

**Published:** 2010-09-30

**Authors:** Benoît Delatour, Stéphane Epelbaum, Alexandra Petiet, Marc Dhenain

**Affiliations:** ^1^CRICM—Team “Alzheimer's and Prion Diseases”, UPMC/Inserm UMR-S 975, CNRS UMR 7225, G.H. Pitié Salpêtrière, 47-83 Boulevard de l'Hôpital, 75651 Paris Cedex 13, France; ^2^NeuroSpin, Bâtiment 145, CEA Saclay, 91191 Gif-sur-Yvette, France; ^3^CNRS, URA 2210, 18 route du Panorama, 92265 Fontenay aux Roses, France; ^4^CEA, I2BM, MIRCen, 18 route du Panorama, 92265 Fontenay aux Roses, France

## Abstract

Identification of biomarkers of Alzheimer's Disease (AD) is a critical priority to efficiently diagnose the patients, to stage the progression of neurodegeneration in living subjects, and to assess the effects of disease-modifier treatments. This paper addresses the development and usefulness of preclinical neuroimaging biomarkers of AD. It is today possible to image in vivo the brain of small rodents at high resolution and to detect the occurrence of macroscopic/microscopic lesions in these species, as well as of functional alterations reminiscent of AD pathology. We will outline three different types of imaging biomarkers that can be used in AD mouse models: biomarkers with clear translational potential, biomarkers that can serve as in vivo readouts (in particular in the context of drug discovery) exclusively for preclinical research, and finally biomarkers that constitute new tools for fundamental research on AD physiopathogeny.

## 1. Introduction

For more than a century, the primary criteria to diagnose Alzheimer's disease (AD) have been relying on clinical observations (development of progressive dementia with a rapid onset of episodic memory impairments). Thanks to the modern refinements of neuropsychological evaluation it is now possible to identify prodromal AD in patients with mild cognitive impairment (MCI) with a specificity of 90% [1]. However, the postmortem examination of brain tissues revealing the pathognomonic lesions of AD (neurofibrillary tangles and senile plaques) is currently the only way to perform a definite diagnosis of this neurodegenerative disease [2]. In vivo biomarkers are gaining ground in bridging the gap between clinical and neuropathological diagnosis of AD. The validation of new AD biomarkers has become a priority to allow an early diagnosis but also to better describe the natural history of the disease [3] and key physiopathological events associated with it [4]. In addition, it is crucial to identify surrogate markers allowing the evaluation of treatment effects and the dissociation between purely symptomatic treatments and disease-modifier actions of the therapies. Today, the most widely used AD markers are based on magnetic resonance imaging (MRI), positron emission tomography (PET), and biochemical exams of body fluids.

The evaluation of brain atrophy markers by structural MRI is the main approach to complement neuropsychological assessment. In particular, medial temporal lobe atrophy is considered to be an excellent criterion for the diagnosis of AD [5]. Volumetric analysis of brain tissues allows to predict conversion from MCI to incipient AD, to dissociate morphological anomalies between patients with early-onset versus late-onset forms of the disease [6, 7] and even to classify the various clinical subtypes of MCI [8]. Cortical hypometabolism quantified by functional MRI (fMRI) or PET is also constantly reported in AD patients, especially in the parietal-temporal regions [9, 10]. It is, however, undoubtedly the direct imaging of AD brain lesions with PET radioligands [11] that has attracted the most successful research opportunities these last years. Various ligands derived from Congo red, thioflavin T, or other molecules showing affinity for aggregated A*β* have been engineered [12, 13]. The use of the PiB (Pittsburgh compound B) ligand described in the original work of Klunk and Engler [14] has received particular attention and has been validated through multicentric studies. This ligand allows the in vivo detection and quantification of brain amyloidosis. PiB has been used to follow the evolution of amyloidosis during disease progression, and to study its relationship with cognitive decline and with disease-modifier treatments [15, 16]. Despite its advantages, PiB presents several limitations: it can display high retention in the brain of aged nondemented subjects [17]; it does not readily detect soluble oligomeric A*β* conformations considered to be highly pathogenic [18]; it shows high affinity for vascular amyloid deposits that may vary from one patient to the other and can be observed in non-AD conditions [19, 20]. In humans, amyloid load, but also tau pathology, can concurrently be evaluated from peripheral markers, for instance, by means of protein dosages in cerebrospinal fluids (CSFs). The combination of various CSF markers (e.g., A*β* 42/A*β* 40 ratio [21], A*β*/tau ratio [22], or tau/phospho-tau ratio [23]) furthermore increases the sensitivity and specificity for AD diagnosis. Recent studies even suggest that amyloid load as detected by PiB-PET and A*β* concentrations measured in the CSF are inter-correlated [24, 25]. CSF dosages can thus be more cost-efficient alternatives to PET examination for the evaluation of amyloid load in patients.

Because of the multiple biomarkers available for the followup of AD brain pathology, recent consensus efforts have led to the conclusion that the diagnosis of AD should first rely on the evaluation of a core clinical criterion (gradual impairment of episodic memory) then on concurrent supportive features, including sets of biological markers (brain atrophy, CSF and plasmatic dosages, cortical hypometabolism, PiB retention, etc.) [26].

Preclinical research, mainly based on the use of mouse models of AD, is an emerging field in the area of biomarkers discovery and validation. Considering the studies performed in humans, the identification and validation of biomarkers in cohorts of patients ironically suffer from the lack of definite diagnosis of AD in the studied subjects. The vast majority of biomarker studies hence rely on the analysis of cognitively normal subjects compared with patients presenting a (high) suspicion of AD dementia. However, some of the controls included in the studied cohorts might present with a preclinical form of AD and display initial neuropathological alterations, albeit not declaring overt detectable cognitive symptoms. Also, the definite neuropathological confirmation of early AD diagnosis in the patients is nearly impossible to obtain in these populations (see however [27]). On the contrary, animal models are specifically engineered to homogeneously reproduce AD brain lesions. This allows to circumvent biases and limitations encountered in human studies that obviously slow down the discovery and validation of new biomarkers of the disease.

**Figure 1:**
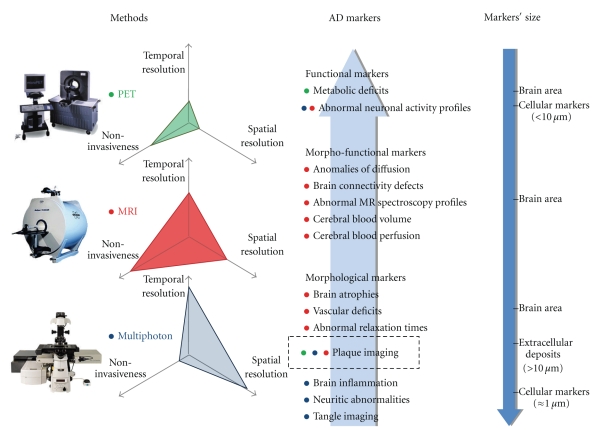
Summary of imaging techniques. This figure schematizes the principal methodologies and tools that can be used to image in vivo the brain lesions and functional alterations developed by mouse models of AD. Radioligands analyzed by PET (green dots) can be used to map brain metabolic deficits in APP(xPS1) transgenic mice. Their relevance for plaque detection in mouse models is more controversial. Applications derived from small animal MR-based imaging techniques (red dots) are more versatile. MRI allows the detection of pathological markers at the regional level (e.g., evaluation of brain atrophy) and also at a microscopic resolution (e.g., visualization of amyloid plaques). In vivo MRI is also a tool to assess functional markers of the pathology such as anomalies in brain perfusion. The recent development of manganese-enhanced MRI has opened new opportunities to map in vivo brain connectivity and neuronal activity with an exquisite spatial resolution. The detection of discrete markers at the (sub)cellular level relies today on the use of high resolution, but also more invasive, imaging techniques that require direct access to brain tissues through a craniotomy in anaesthetized animals: with multiphoton microscopy (blue dots) it is for instance possible to detect intraneuronal neurofibrillary tangles as well as dystrophic neurites in APP(xPS1) transgenic mice.

Here we will review the most widely used AD biomarkers and their associated techniques that can be studied or deployed in animal models (see Figure 1). We will focus on morphological and microscopic biomarkers of AD as well as on functional markers. The relevance/importance of the preclinical studies on AD biomarkers will be discussed. Many rationalizations will be explored in parallel throughout this paper.

1. First, the discovery of new biomarkers in AD animal models can be implemented with a translational aim with the hope that it will ultimately pave new ways for practical applications in human patients. Indeed, we still need to refine the existing markers of AD and concurrently identify new biomarkers.
2. In parallel, in vivo neuroimaging of biomarkers in AD animal models can provide crucial information to better understand the disease processes. For instance, the analysis of AD models might help disentangling the temporal relationship between the neuropathological lesions themselves and deciphering their impact on neuromorphology and on brain function. Advances in our knowledge of AD physiopathogeny have hence been associated with the development of new preclinical imaging techniques applied to AD transgenic mice, such as in vivo two-photon laser scanning microscopy [28].
3. Finally, preclinical biomarkers can also be used to follow up animal models during drug evaluation.

## 2. Imaging of Brain Anatomy in AD Mouse Models

In humans, brain atrophy is one of the best-established markers for the diagnosis and prognosis of AD. Detecting and quantifying the process of atrophy in the rodent brain might be viewed as a challenge as the mouse brain is approximately 3000-fold smaller than the human cerebrum. The use of high-field MRI has nevertheless allowed bypassing these technical limits. In vivo analysis of brain morphology in preclinical models allows to longitudinally monitor the possible onset and progression of localized atrophies [29] and also to visualize the effects of disease-modifier treatments [30].

The preclinical study of brain atrophy in AD animal models has been mainly performed in transgenic mouse lines carrying familial AD mutations (APP and/or PS1-2 genes) and developing brain amyloidosis. The first reports were obtained in the PDAPP model and demonstrated that these transgenic mice overexpressing mutant APP display a severe atrophy of the medial temporal lobe with a focus on the hippocampus [31–36]. These original observations, derived from in vivo experiments and/or from postmortem analysis, suggested a similarity between morphological anomalies in AD patients and in mouse models; however, other data strongly argue against this initial statement.

1. Brain atrophy as described in AD transgenic lines, have multiple foci and can involve brain areas that are not usually atrophied in AD patients. For instance, posterior subcortical atrophy (mesencephalon) is displayed in APPxPS1 transgenic mice [37]. Also, white matter anomalies (including fiber tract atrophy in different regions: corpus callosum, anterior commissure, dorsal hippocampal commissure, fornix, corticospinal tract, etc.) have been described in various AD transgenic lines [37–39]. These neuropathological alterations encountered in AD mouse models, especially those affecting callosal fibers [33] are hardly evocative of human pathology, both in qualitative and in quantitative aspects.
2. Moreover, in some mouse lines, the hippocampal atrophy of AD transgenics appears to have a very early onset during ontogenesis, which contrasts with the slow and gradual atrophy process depicted in AD patients. The data obtained in APP(xPS1) mice might thus underpin the occurrence of developmental abnormalities rather than of the true age-dependent AD-like brain atrophy [35]. Furthermore, the atrophy of the hippocampus is not constantly reported in AD transgenics. In a particular severely affected APPxPS1 model we demonstrated that there was no significant volume reduction of this brain area, even in the oldest animals [37]. In this study, the lack of hippocampal atrophy was further confirmed by postmortem histological assessment. In addition, two recent MRI studies performed on other APPxPS1 transgenic mice depicted a paradoxical accelerated growth of the hippocampus in the transgenics as compared to the wild-type animals [39, 40].

These puzzling data have to be reinterpreted in the context of the amyloid cascade hypothesis. The major tenet of this physiopathological hypothesis is that the deposition of A*β* in brain parenchyma precedes and triggers subsequent brain anomalies including tissue loss and atrophy. Observations in APP(xPS1) transgenic mice somewhat dispute this belief: atrophy can be observed before the onset of brain amyloidosis or can concurrently be absent despite a heavy local amyloid burden. In addition, no correlations between amyloid load and overall brain volumes have been highlighted [37].

Brain volumetry, even when assessed in vivo, is thus a questionable biomarker in AD APP(xPS1) transgenic mice. Therefore, one has to be cautious when using brain volumes as morphological readouts for preclinical research as it has become clear that brain tissue atrophies are qualitatively and quantitatively different in mouse models of brain amyloidosis and in AD patients. This obviously constitutes a pitfall for subsequent translational efforts.

## 3. Imaging of Brain Microscopic Lesions in AD Mouse Models

### 3.1. Indirect Detection of Cerebral Amyloidosis

APP(xPS1) mouse lines are an adequate model to develop new imaging techniques in order to follow up onset and progression of amyloid burden. As a first step, global MR parameters expected to reflect A*β* deposition have been explored. For example, a reduction in T2 relaxation time has been depicted in APPxPS1 mouse lines [41, 42]. Such reduction in T2 is presumably explained by A*β* accumulation as stresses the correlation between T2 and histologically-assessed local amyloid load [43]. There is, however, an old controversy whether similar abnormal relaxation times can be depicted in human AD brains or not (see [44] but [45, 46]), which sheds some doubts on the possibility to extrapolate preclinical findings to the development of new valid diagnostic tools for human patients.

### 3.2. Direct Imaging of Plaques (and of Other Microscopic Lesions) 

#### 3.2.1. In Vivo Multiphoton Microscopy Imaging

In transgenic mice, in vivo multiphoton microscopy is a brain imaging modality that allows to image amyloid deposits and associated lesions in small tissue volumes at very high resolution (1 *μ*m) (for reviews of the methods, see [28, 47]). After surgery involving local craniotomy [48] a fluorescent dye (e.g., thioflavin S or methoxy-X04) can be administered peripherally to label plaques and visualize them through the skull open window, using nondestructive multiphoton laser excitation. The field of view of the technique is limited, but images can be efficiently acquired from cortical surface up to 800 *μ*m of depth. Visualization of vascular arborizations (intravenous injection of a fluorescent dye such as Texas red dextran) is generally performed simultaneously to provide constant landmarks for repeated imaging. Multiphoton imaging methods have also been successfully applied to visualize neurofibrillary lesions in vivo. For example, Spires-Jones and coworkers labeled in vivo the NFTs of rTg4510 mice, a model of taupathy, by application of thioflavin S on brain surface after dura resection [49, 50].

In vivo multiphoton microscopy has significantly increased our knowledge on AD physiopathogeny. Indeed, the method allows in vivo longitudinal studies (across weeks or months) of the kinetics of plaque progression [51, 52] and measurements of the effects of treatments (e.g., immunotherapies) on plaque clearance [53]. Using these methods, Meyer-Luehmann and colleagues [54] suggested that the initial formation of plaques might be an acute event (plaques were observed to form quickly within 24 hours). However, these observations have recently been challenged by another report indicating that amyloid plaques grow more slowly over periods of weeks [55]. Analysis of the role of brain inflammation in AD pathogeny has also been clarified by in vivo multiphoton imaging. For instance, the relationship between plaques (labeled with Methoxy-X04) and activated microglia (overexpressing GFP through an Iba-1 promoter) has recently been described in APPxPS1 transgenics [56]. In a smart-engineered model (GFP inserted in the CX3CR1 chemokine receptor of the 5xTg AD model, which also overexpressed Thy1-driven YFP), Fuhrman and colleagues were able to describe the live dialogue between microglia and neurons in the presence of AD lesions [57]. Finally, in vivo multiphoton microscopy studies have also allowed investigating the local toxicity of A*β* deposits on surrounding synapses and on neuritic processes. In direct support to the amyloid cascade hypothesis, Meyer-Luehmann et al. [54] demonstrated that plaques create a local microenvironment (a reservoir of bioactive molecules) that gradually promotes pathological changes in neighboring neurites within days. Koffie et al. [58] underlined that A*β* oligomers, the so-called “modern culprits” of AD, might be crucial players in these synaptic/neuritic alterations; in APPxPS1 mice they labeled plaques with Methoxy-XO4 and oligomers by infusing the antioligomers NAB61 antibody conjugated to a fluorescent dye on the cortex after dura opening. They were able to detect in vivo a hallo of oligomers surrounding senile plaques; subsequent histological analysis underlined that this peripheral hallo was associated with a severe synaptotoxic effect (loss of PSD75 immunoreactivity). AD-related neuritic anomalies detected by in vivo multiphoton microscopy might also serve to evaluate the impact of new therapies. For instance, it has been demonstrated that antioxidant treatments have a rescuing action on neuritic dystrophies (abnormal neurite curvature of fluorescently labeled neurons) while not reducing A*β* plaque size [59]. On the contrary, the same research group provided data showing that treatment with one *γ*-secretase inhibitor (LY-411575) does not affect the neuritic defects of APPxPS1 transgenics [60].

#### 3.2.2. PET Imaging of Amyloid Plaques

Although in vivo multiphoton microscopy provided very interesting results on the physiopathogeny of AD, the method cannot be easily translated to patients. In humans, the best-established method to detect amyloid plaques relies on the use of the PiB radioligand. Although this compound was initially evaluated in transgenic mouse models of AD [61] to detect amyloid lesions by multiphoton imaging, it did not lead to conclusive results when used to detect amyloid plaques by PET in APPxPS1 transgenic mice [62, 63]. Also, it has been evidenced that 18F-FDDNP, a good radioligand for amyloid plaques and tangles in humans, does not show good retention in the brain of Tg2576 mice [64]. Using ultrahigh specific-activity PiB (200 GBq/mole; injection of the tracer immediately after synthesis), Maeda and collaborators were nevertheless able to show increased retention of the ligand in APP transgenics. It is hence possible that paucity of high-affinity PiB binding sites in APP(xPS1) transgenic mice is the explanation for previous imaging failures. It might also be possible that the density of the high-affinity PiB binding sites varies from one transgenic line to the other (Maeda and collaborators used the APP23 line while previous works of Klunk and Tomaya were performed in Tg2576 APP mice and in APPxPS1 transgenics derived from the Tg2576 model). Interestingly, Rosen et al. recently demonstrated that PiB-binding is also reduced in aged rhesus macaques, chimpanzees, and squirrel monkeys despite the fact that the nonhuman primates they evaluated displayed heavy amyloid burden [65]. It can be tentatively concluded that PiB is an efficient probe only for human-specific molecular components of A*β* deposits. Despite differences between humans and animals, the development of new radioligands for AD brain lesions still strongly relies on preclinical tests in APP(xPS1) transgenic mice. For example, the recently-developed AV-45 ligand, which is expected to become a good substitute to PiB in the coming years, has first been tested in transgenic mice [66].

#### 3.2.3. MR Imaging of Amyloid Plaques

Direct noninvasive in vivo imaging of plaques in APP(xPS1) transgenic mice is a very stimulating research area and the MRI-based detection of amyloid deposits is actively pursued as an alternative technique to PET imaging in preclinical research. Numerous studies performed in APP(xPS1) transgenic mice underlined that discrete aggregated A*β* deposits locally modify MR contrasts. These contrast anomalies can be detected in postmortem conditions (extracted brains) that favor the acquisition of high-resolution images [67–70] but they can also be detected in vivo in anaesthetized mice [71–73]. One mechanism that may explain the MRI spontaneous contrast of plaques is the accumulation of iron in amyloid deposits that locally alters the relaxation time of tissues [41, 74, 75]. The recent work of Meadowcroft and collaborators [76] emphasized that both the high iron concentrations and the interaction of water with dense aggregated amyloid mass can concomitantly modify transverse relaxation rates but that these two mechanisms might be differentially involved in human versus in mouse brain tissues.

MR imaging of plaques in rodents can also be performed using specific probes. For example, the amyloid-binding styrylbenzenes derived from the Congo red dye and complexed to 19F (fluorine) can pass through the blood-brain barrier (BBB). One publication suggested that these derivates can allow detection of A*β* deposits in living Tg2576 mice [77]. However, these results have not been replicated. A*β* conjugated to gadolinium or monocrystalline iron oxide nanoparticles (MIONs) are more robust methods for amyloid plaque visualization. When administered peripherally (intravenous or intracarotid injection), the peptide-contrast agent complexes have some tropism for amyloid plaques thanks to the binding affinity of A*β* peptides for other A*β* peptides. However, brain penetration of the conjugates often requires the opening of the BBB (using mannitol) or the complexion with polyamines (e.g., putrescin). These vectorized contrast agents can be used “in vivo” to detect amyloid plaques of AD transgenics [78, 79] and even to quantify amyloid load on a voxel-based analysis [80]. Recent studies emphasized the use of small-sized antibodies to build efficient MRI probes against A*β* deposits. For instance, Ramakrishnan and collaborators used polyamine-modified Fab fragments complexed to gadolinium to perform ex vivo amyloid plaque visualization by MRI in APPxPS1 mice [81]. Also, the group of Beka Solomon engineered anti-A*β* ScFv antibodies by phage display, mainly with a (immuno)therapeutical aim [82]. Interestingly, these small antibodies, when infused intranasally, have the capacity to penetrate into the brain and to reach the amyloid plaques, not only at the injection loci (olfactory bulbs) but also at long distances from the nose, for instance, in the hippocampi [83]. It might hence be predicted that complexing these phage-ScFv with an MRI contrast agent would allow in vivo plaque detection. Also, the small homodimeric antibodies of camelidae have been shown to be able to cross the BBB after intracarotid infusion and to reach their targets in brain parenchyma [84]. Some of these antibodies, much smaller than the classical IgGs, have been specifically produced to recognize A*β* epitopes [85, 86] and might be valuable tools to initiate new strategies for in vivo plaque detection.

The MRI-based methods implemented in rodents could find a first application in drug development as they offer a solution to monitor online the disease-modifier effects of treatment in animal models of AD. Up to now, it has been difficult to evaluate the translation from these preclinical data obtained in mice to valid MRI protocols utilizable in AD patients. However, the rising development of high-field MRI for clinical and research uses might accelerate the implementation of such noninvasive methods. Preliminary studies hence suggest that amyloid plaques will soon be detectable by MRI in humans [87, 88].

#### 3.2.4. Other Imaging Approaches to Detect Amyloid Plaques

Alternative methods for plaque imaging are under scrutinization in APP(xPS1) mice (e.g., Near InfraRed Fluorescence (NIRF) in vivo imaging of new compounds that bind to plaques [89] or Diffraction Enhanced Imaging (DEI), a phase contrast X-ray imaging technique that provides high soft tissue contrast which allows A*β* plaque detection [90]). These methods might also find applications as preclinical tools to screen the effects of interventional therapies.

#### 3.2.5. Relevance of Amyloid Plaque Detection

To conclude on amyloid plaque imaging, it is important to remind that while research efforts have contributed to implement various imaging techniques for the in vivo detection of amyloid plaques in animal models, the exact status of these lesions as core biomarkers of AD is still obscure and largely discussed. It has been known for decades that A*β* accumulation can be observed in nondemented human individuals and this finding has recently been confirmed by PiB-PET scans that identified a subset of intellectually normal persons with a high PiB fixation indicating severe A*β* accumulation [17]. In addition there is, today, no conclusive evidence to support the hypothesis that reducing amyloid load by stimulating plaque clearance has any clinical effects in AD patients [91]. Hence, the relevance of imaging plaques as primary readouts for disease detection/progression as well as for the evaluation of disease-modifier therapies is challenged if not questionable. This urges the need for (1) better refining the status of amyloid plaques as in vivo gold standard markers of the disease, (2) stimulating new preclinical research efforts towards alternative biomarkers of AD.

### 3.3. Imaging of AD-Related Alterations of Brain Microstructure

Different imaging parameters are altered in animals developing cerebral amyloidosis but not necessarily as a direct consequence of A*β* accumulation. For example, anomalies of water diffusion detected by in vivo MRI have been described in AD transgenics accumulating amyloid plaques [92–94] (see however [95]). The exact origin of these diffusion defects is still uncertain (highly hydrophobic deposits might cause constraints on water diffusion but these defects might also come from loss or alterations of the white matter). MR spectroscopy shows a decrease of N-acetylaspartate (NAA) in the brains of APPxPS1 transgenics [96]. NAA is considered to be a good marker of neuronal viability; its decrease in APP(xPS1) mice might be caused by the intraparenchymal accumulation of A*β*; some reports indicated indeed a negative correlation between amyloid load and NAA levels [97]. Although of interest, these methods have not been used to followup disease progression in AD preclinical models, maybe because the tracked markers are not specific to AD condition and can be detected only in the oldest animals. It is uncertain whether therapeutical evaluation of disease-modifier treatments could rely on such markers.

## 4. Imaging of Brain Dysfunction in AD Mouse Models

Besides directly imaging the microscopic and macroscopic lesions of AD, it is also possible, even in animal models, to follow the evolution of the pathological process through the brain functional disorganizations it promotes (e.g., perfusion and neuronal activity impairments).

### 4.1. Vascular-Perfusion Anomalies

Since the initial observation of the occurrence of cerebral amyloid angiopathy in AD patients, it has been well established that vascular abnormalities are a major phenotype of the disease. Using dedicated postmortem morphological analysis (e.g., corrosion casts) similar vascular impairments have been described in AD transgenics under the form of eliminated/truncated brain vessels or dysmorphic vascular architecture [98, 99]. Importantly, such drastic changes in the morphology of brain vessels can also be visualized using in vivo magnetic resonance angiography (MRA) that allows the detailed detection of the vascular arborization in the brain. MRA has been successfully applied to APP [98] and APPxPS1 [100] mice that both demonstrate abnormal arterial voids. These methods are interesting tools to measure the preventive/curative effects of vascular-oriented therapies although the relationship between the incidence of vessels anomalies and A*β* brain deposition remains to be clarified.

In parallel, the cerebral blood volume (CBV) of AD transgenics has been observed to be reduced (for recent illustration in a triple APPxPS1xtau line, see [101]). Shrinking of the cerebrovascular space in rodents can be estimated in vivo using MR detection of susceptibility contrast agents such as gadolinium administered intraperitoneally [102] or monocrystalline iron oxide nanoparticles injected intravenously [103]. Restoration of normal CBV maps in AD transgenics has been demonstrated across therapeutic assays (e.g., long-term treatment with nonsteroidal anti-inflammatory drugs in Tg J20 mice [102]).

As a more functional consequence of vascular impairments in AD transgenics, blood perfusion is also decreased in different mouse models. This perfusion decrease can be assessed using standard autoradiographic methods [104], by laser Doppler [105], and by in vivo MRI (e.g., by means of spin-labeling techniques). For instance, MRI detection of perfusion impairments has been performed in APPxPS1-Ki mice [106] and in PS2APP mice [107].

### 4.2. Metabolic, Cellular, and Network Dysfunction

Recordings of cortical hypometabolism as measured by PET imaging and brain hypoactivities assessed by functional MRI are hallmarks classically described in AD patients. Similar anomalies can be observed in mouse models of AD. Thanks to their high spatial resolution, autoradiographic methods (e.g., of fluorodeoxyglucose-FDG or of 2-deoxy-D-glucose) have been applied to map brain metabolic activities in PDAPP mice [108, 109] and in APPxPS1 transgenics [110]. In these transgenic mice, hypometabolism was constantly evidenced in the association cortex (e.g., posterior cingulate cortex), evocative of findings obtained in AD patients. However, the relationship between these functional anomalies and amyloid burden is still questioned [109].

In vivo metabolic imaging in mice undergoes rapid development but it is technically challenging because PET imaging in rodents is limited by its low resolution, the cost of the ligands, and access to micro-PET devices. Also, recent data indicated no differences in FDG-PET between AD transgenics (Tg2576 mice) and control animals [64].

Alternative ways to visualize brain activity in living mice are available. The recent development of in vivo multiphoton calcium imaging techniques applied to AD mouse models [111–113] has provided us with remarkable new findings on the disease physiopathogeny but their use for therapeutic preclinical development is doubtful. Manganese-Enhanced MRI (MEMRI) is a better-established method to follow brain activity in rodents. It allows the in vivo detection of active neuronal populations in rats, mice, and in nonhuman primates [114]. Manganese is an analog of calcium and after peripheral or stereotaxic administration it can penetrate into brain neurons via voltage-dependent Ca2+ channels. Paramagnetic properties of manganese allow its direct visualization by MRI. A raise in manganese concentrations has been described in selected brain regions following interoceptive/exteroceptive stimulations [115, 116]. We are not currently aware of any reports that have applied this pioneering method to map brain activities in APP or APPxPS1 mice. Kimura and collaborators conversely used MEMRI analysis in mice overexpressing tau and accumulating hyperphosphorylated tau in their brains [117]. The authors reported clear cortical hypoactivation in the tau mice, at the level of the postrhinal cortex (homologous to the parahippocampal area of primates, including humans) and in tight association with the occurrence of visuospatial cognitive deficits. In addition to being a good marker of activated neurons, manganese is also transported in axons via microtubules. It can therefore be used as an in vivo transsynaptic tracer to label brain networks in situ. Delays in manganese transport, assessing malfunction of brain connectivity, presumably as a response to synaptic/neuritic underlying pathologies, have recently been evidenced in APP and APPxPS1 transgenic mice [118, 119]. Interestingly, the analysis of transneuronal manganese transport can be used to screen the effects of therapies against AD (e.g., memantine [120]). Although associated with known putative toxic effects [114, 121], MEMRI might rapidly become a prominent method for the analysis of brain dysfunction in AD transgenic mice and of functional recovery after therapeutical intervention.

## 5. Conclusions

AD biomarkers have the opportunity to be developed, tested and validated in mouse models of the disease. In many cases, mice have allowed to produce the first proof of concept for biomarkers that have been then translated to humans. This is the case of radioligands that now allow the detection of amyloid plaques by PET in patients. MR biomarkers of amyloid plaques underwent a similar development pipeline (first tested in mice, then applied to humans). In the near future, one can predict that transgenic models will play a decisive role in the investigation of new biomarkers of AD pathology, such as, for example, markers of Tau pathology that are critically missing today.

Interestingly, major brain changes detected in AD patients (e.g., cerebral atrophy or reduced brain metabolism detected by FDG-PET) are not always replicated in animals. Such discrepancies can be explained by technical considerations but are also attributable to the intrinsic characteristics of the model and to its inability to mimic all aspects of the human pathology. On the other hand, a large range of biomarkers detecting AD brain alterations in humans is detectable in a similar way in transgenic mice. For instance, biomarkers of amyloid plaques, perfusion, and cerebral blood volume are all valid both in AD patients and in mouse models of the disease. These markers are critical to followup disease progression, to monitor the effects of treatments during preclinical studies, and to predict possible therapeutic effects in humans. They can thus be considered as good translational biomarkers of AD pathology. Finally, some markers and their associated methodologies have been implemented in animals and will probably be restricted to preclinical studies. These markers could be of importance to refine our understanding of the physiopathogeny of the disease or, alternatively, to detect the effects of new therapies initiated in animal models. This is particularly true for invasive approaches such as bi-photon imaging of amyloid plaques or for methods relying on potentially toxic contrast agents such as manganese-enhanced MRI.

To conclude, transgenic mouse models of AD can be used as tools to identify and validate new translational biomarkers of AD pathology. In parallel, the markers identified in these models might have a field of application restricted to the sole preclinical research but still with an interest for both fundamental and therapeutical investigations.
